# Exhausted CD8+T Cells in the Tumor Immune Microenvironment: New Pathways to Therapy

**DOI:** 10.3389/fimmu.2020.622509

**Published:** 2021-02-02

**Authors:** Weiqin Jiang, Yinjun He, Wenguang He, Guosheng Wu, Xile Zhou, Qinsong Sheng, Weixiang Zhong, Yimin Lu, Yongfeng Ding, Qi Lu, Feng Ye, Hanju Hua

**Affiliations:** ^1^ Department of Colorectal Surgery, The First Affiliated Hospital, Zhejiang University, Hangzhou, China; ^2^ Department of Radiology, First Affiliated Hospital, Zhejiang University School of Medicine, Hangzhou, China; ^3^ Department of Pathology, First Affiliated Hospital, College of Medicine, Zhejiang University, Hangzhou, China; ^4^ Department of Surgical Oncology, First Affiliated Hospital, College of Medicine, Zhejiang University, Hangzhou, China; ^5^ Department of Medical Oncology, First Affiliated Hospital, College of Medicine, Zhejiang University, Hangzhou, China; ^6^ College of Medicine, Zhejiang University, Hangzhou, China

**Keywords:** CD8+ T cell exhaustion, CD8+ T cell activation, differentiation, immunotherapy, tumor microenvironment

## Abstract

Tumor-specific CD8^+^T cells are exposed to persistent antigenic stimulation which induces a dysfunctional state called “exhaustion.” Though functioning to limit damage caused by immune response, T cell exhaustion leads to attenuated effector function whereby cytotoxic CD8^+^T cells fail to control tumor progression in the late stage. This pathway is a dynamic process from activation to “progenitor exhaustion” through to “terminally exhaustion” with distinct properties. With the rapid development of immunotherapy *via* enhancing T cell function, new studies are dissecting the mechanisms and identifying specific biomarkers of dynamic differentiation during the process of exhaustion. Further, although immune checkpoint inhibitors (ICIs) have achieved great success in clinical practice, most patients still show limited efficacy to ICIs. The expansion and differentiation of progenitor exhausted T cells explained the success of ICIs while the depletion of the progenitor T cell pool and the transient effector function of terminally exhausted T cells accounted for the failure of immune monotherapy in the context of exorbitant tumor burden. Thus, combination strategies are urgent to be utilized based on the reduction of tumor burden or the expansion of the progenitor T cell pool. In this review, we aim to introduce the concept of homeostasis of the activated and exhausted status of CD8^+^T cells in the tumor immune microenvironment, and present recent findings on dynamic differentiation process during T cell exhaustion and the implications for combination strategies in immune therapy.

## Introduction

Cytotoxic CD8^+^T cells (CTLs) are a major population of immune cells that control and clear tumor cells. CTLs need to be primed and activated first, and then hone to tumor site to induce an efficient immune response (1). However, due to immunotolerance and immunosuppression mechanisms, these T cells are often sub-optimally primed and differentiate into a dysfunctional state called “exhaustion,” thus failing to control tumor growth, leading to tumor progression. Multiple extrinsic and intrinsic factors have been put forward to account for the possible mechanisms, among which co-inhibitory receptors are thought to be one of the major mechanisms impairing T cell effector function (2).

In the past decade, novel checkpoint blockades have made a great breakthrough in treating multiple solid cancers. Antibodies targeting inhibitory receptors including cytotoxic T lymphocyte-associated Antigen 4 (CTLA-4) and programmed cell death 1 (PD-1) successfully relieve inhibition and enhance T cell effector function, leading to improved clinical efficacy in treating several solid tumors (3), including advanced melanoma, non-small-cell lung cancer, renal cell carcinoma and metastatic bladder cancer (4). Despite its great clinical success, most patients do not experience complete response. Patients who do not respond to initial PD-1/PD-L1 blockade are referred to as having “primary resistance” to therapy (5). Furthermore, a growing subset of patients develop “acquired resistance” to immunotherapy, which is defined as a clinical scenario whereby a cancer initially responds to immunotherapy, but after a period of time, relapses and progresses (5). In clinical practice of using immune checkpoint blockade, most patients still show limited efficacy with either a poor response or a transient reinvigoration soon to be resistant, necessitating the understandings of mechanisms of resistance and exploring corresponding strategies to overcome the resistance.

In this review, we aim to introduce new understandings of inhibitory receptors beyond exhaustion, providing new insights into checkpoint blockades treatment. Further, we highlight the dynamic differentiation during T cell exhaustion and discuss the implications for combination strategies in immune therapy.

## Homeostasis of the Activated and Exhausted Status of CD8^+^T Cells in the Tumor Immune Microenvironment

### T Cell Exhaustion in the Tumor Microenvironment Is a Special Hyporesponsive State

When naïve CD8^+^ T cells encounter antigen during an acute infection, they are activated and then differentiate into cytotoxic effector T cells that control and even clear the pathogen/antigen. Once the pathogen/antigen has been eliminated, most effector T cells undergo apoptosis while a minority survive and differentiate into memory T cells which function as a back up to fight against subsequent infection (6, 7). However, in face of persistent antigen stimulation in chronic virus infections or tumors, T cell differentiation is found to derail toward a special hyporesponsive state namely “exhaustion.”

T cell exhaustion is defined as progressive loss of effector function (loss of IL-2, TNF-α, and IFN-γ production) and sustained expression of inhibitory receptors such as PD-1, T cell immunoglobulin domain, and mucin domain-containing protein 3 (Tim-3), CTLA-4, lymphocyte-activation gene 3 (LAG-3), and CD160 with a transcriptional program distinct from functional effector or memory T cells (6). Tolerance, anergy and exhaustion are several terminologies used to describe hyporesponsive T cells. Tolerance refers to the main mechanism to prevent autoimmunity by central/peripheral inactivation of self-reactive T cells (7, 8). Anergy describes incompletely activated T cells with absent co-stimulatory signals and/or high co-inhibitory stimulation (9, 10). Among those, exhaustion was especially put forward as a term to describe a functional but hyporesponsive state having undergone initial activation in the context of chronic infection or tumor, distinguishing it from tolerance and anergy.

Exhaustion was observed in a chronic infection model of lymphocytic choriomeningitis virus (LCMV) strain clone 13 and later demonstrated in a tumor microenvironment (11, 12). In chronic viral infection, virus-specific T cells initially acquire effector function, and driven by chronic viral antigen stimulation, progressively lose effector function in a hierarchical manner firstly through loss of proliferative ability and IL-2 production, then loss of TNF-α production and finally loss of IFN-γ production (13). Duration of activation impacts the ability of CD8^+^T cells to secrete pro-inflammatory cytokines and elaborate cytotoxic function (2, 14). Though sharing common features in reduced immune function, exhausted T cells in the tumor microenvironment are distinct from those in a chronic infection (15).

Tumorigenesis is a long-term process during which interactions between tumor cells and immune system remodel the tumor microenvironment and change differentiation of CD8^+^T cells. The progressive loss of T cell function in cancer is mainly impeded by three stumbling blocks (16). Firstly, during thymic maturation, partial tumor-specific T cells are depleted because many tumor cells display self-antigens and self-tolerance mechanisms negatively select them. Due to a “leaky” immune tolerance mechanism, considerable numbers of self/tumor-specific T cells still survive with low affinity for antigen recognition as compared to virus-specific T cells (15, 16). Moreover, antigen-presenting cells (APCs) are weakly activated due to a lack of innate stimulators in the special non-inflammatory context, resulting in the suboptimal activation of tumor-specific T cells (17). The remaining stumbling block is the induction and maintenance of T cell hyporesponsiveness by the special immunosuppressive tumor microenvironment (TME). Cancer immunoediting describes the dual host-protective and tumor-promoting roles of immunity (18). While the immune system eliminates tumor cells as immune surveillance, tumor cells also recruit immunosuppressive cells and secrete related inhibitory factors to generate the immunosuppressive tumor environment and persistently suppress T cell immune function with increasing tumor development (19).

Thus, despite the fact that CD8^+^ cytotoxic T cells play a pivotal role in eliminating tumor cells, they often differentiate into an exhaustion state and fail to control tumor progression in the late stage. While sharing some common features with exhausted T cells in chronic viral infection, tumor-specific exhausted T cells display distinct properties due to immunotolerance and immunosuppression mechanisms and effective methods to reinvigorate them will significantly impact the progression of tumor.

### Molecular Signatures of Activation and Exhaustion Are Intertwined

T cell exhaustion arises in the face of persistent T cell activation which may explain that surface markers and transcriptional signatures of exhausted T cells are intertwined with activated T cells (20). Both exhausted and activated CD8^+^ T cells up-regulate genes related to cell cycle activation, T cell homing and co-inhibitory receptors which down-regulate memory related genes (21, 22). However, as exhaustion results in the failure to control the tumor in the late stage, specific markers for exhaustion are demanding to be identified as target sites to specifically reverse dysfunctional T cells.

Inhibitory receptors are hallmarks of dysfunctional CD8^+^T cell which are upregulated on naïve T cells undergoing activation and differentiation (23). The breakthrough in immune checkpoint blockade therapy (ICB) has raised great attention in identifying the underlying inhibitory receptors and their clinical significance. It is well acknowledged that multiple and high expression of inhibitory receptors such as PD-1, Tim-3, CTLA-4, LAG-3, T cell immunoreceptor with immunoglobulin, and ITIM domains (TIGIT), B and T lymphocyte attenuator (BTLA), 2B4 and CD160 are highly associated with the severity of the dysfunction phenotype (2). There are several mechanisms accounting for dampening T cell activation and leading to the dysfunction, including blocking downstream co-stimulatory signals, restraining metabolic changes, interfering the proliferation or suppressing inflammatory factors (24). However, in recent years the implication of inhibitory receptors is thought to be more than exhaustion (25).

In a “tide model,” the expression of co-signaling molecules including co-inhibitory receptors and co-stimulatory receptors is differentially and tightly regulated by signals involved in T cell activation and differentiation, where inhibitory receptors are up-regulated in order to counterbalance co-stimulatory signals following the peak of activation (26). With a primary signal to initiate immune response, stimulatory and inhibitory signals follow to cooperate to induce an inflammatory response and limit damage to the surrounding tissue. Thus, inhibitory receptors are also found upregulated even in physiological immune process and function as a mechanism to balance activity of immune cells and ensure immune homeostasis (14). For instance, it was previously thought that PD-1 regulates T cell dysfunction in chronic infection and tumor control whereby PD-1^hi^ cells exhibit an intense exhausted gene signature (27). However, PD-1^hi^ CD8^+^ T cells in healthy humans are not significantly correlated with the PD-1^hi^ exhausted gene signatures of LCMV-specific CD8^+^T cells from mice, while PD-1 expression does not directly affect cytokines secretion of CD8^+^T cells (28). PD-1 was found to be upregulated in recently activated effector cells (22). Further, PD-1^+^CD8^+^ T cells in peripheral blood mononuclear cells (PBMC) of melanoma patients are also found not to be necessarily functionally impaired (29). Moreover, the absence of PD-1 does not reverse T cell dysfunction but conversely promotes accumulation of terminally exhausted T cells during tumorigenesis (30).

The overlap of inhibitory receptors between dysfunction and activation thus complicates the identification and development of effective targeted therapies. An activation-dependent exhaustion program whereby exhaustion of gene expression is highly correlated with the expression of both cytotoxicity markers and T cell activation states (22, 25, 31), and thus expression of coinhibitory receptors may not be sufficient to distinguish T cell activation from exhaustion. Consequently, it is challenging to discover specific markers indicative of the dysfunctional T cell state. Recently, a pattern of chromatin accessibility enriched for consensus motifs for Nr4a and NFAT transcription factors was specifically associated with T-cell exhaustion (32). The overexpression of Nr4a1 was found to inhibit the differentiation of effector T cell but induce T cell tolerance (33). At the meanwhile, depletion of Nr4a transcription factors reversed the dysfunctional state of T cells (33, 34). These studies imply that Nr4a transcription factors induce the differentiation of exhausted T cells while inhibit the effector function. Driven by chronic T cell receptor stimulation and NFAT activation, expression of the nuclear factor TOX is upregulated in dysfunctional T cells (35). However, *Tox*-deleted TST cells remained dysfunctional without upregulation of inhibitory receptors (such as PD-1, CD39, Tim-3, 2B4, and TIGIT) and failed to persist in tumors (35). Hence, TOX-induced gene regulation of inhibitory receptors and other exhaustion-associated molecules may function to prevent overstimulation and activation-induced cell death (35). In addition, enrichment of a gene model containing *Tox* distinguishes progenitor exhausted CD8^+^ T cells (as outlined below) in chronic infections from memory precursor cells (36). TOX deficiency leads to loss of progenitor-like CD8^+^ T cells and reduces persistent resistance to pathogen of (36, 37). This finding suggests that TOX may drive T cells differentiation toward progenitor-like CD8^+^ T cells and the absence of TOX results in reduced capacity to generate exhausted T cells and thus the failure of a persistent immune response.

Furthermore, by using single-cell RNA-seq, activation and dysfunction gene modules can be separated at the single-cell level (20). Intracellular metallothioneins (MT1 and MT2) that regulates zinc metabolism was found highly enriched in dysfunctional CD8^+^ tumor-infiltrating T cells (TILs) at the same time as targeted deletion of metallothioneins reversed T cell dysfunction and controlled tumor growth without reduction of expression of co-inhibitory receptors (20). It reinforces the concept that co-inhibitory receptors may play a significant role in an activation-associated transcriptional program, but differs from the program driving dysfunction in CD8^+^ T cells. By analyzing the RNA profiles of TILs from wildtype and MT1/2 deficient mice, a separate ranking of genes by their association with activated and dysfunctional T cell phenotypes was obtained to define four separate modules including: (1) activation (but no dysfunction), (2) dysfunction (but no activation), (3) activation and dysfunction and (4) neither (a “naïve/memory-like” module) (20). It provides us with a new gene model that is expressed specifically in dysfunctional T cells but not in activated T cells to develop targeted therapy specific for the dysfunctional T cell state.

To fulfill the effective anti-cancer immune response, a series of stepwise events named as “the Cancer-Immunity Cycle” must be fulfilled at every point (1). The Cancer-immunity Cycle consists of several steps. First of all, encountering antigens on activated dendritic cells result in the priming and activation of CD8^+^ T cells resulting in expansion and differentiation into cytotoxic T cell (CTLs). These CTLs then circulate *in vivo*, extravasate at inflammatory sites, penetrate into the tumor tissues and finally recognize and kill tumor cells (1, 38). And any conditions that forestall this process at any step can lead to the failure of immune response. The goal of cancer immunotherapy is to initiate or reinitiate a self-sustaining cycle of cancer-immunity with restrained autoimmune inflammatory responses. Though the development of immune checkpoint blockades has achieved great success in cancer therapy, the perturbation to immune exhaustion can provoke inappropriate autoimmune reactions in multiple tissues including the skin, intestine, liver and lung (39). The most common autoimmune-like immune-related adverse events (irAEs) after checkpoint blockades are dermatologic (47%–65%), colitis (30%–48%), hepatitis (5%–30%), and/or endocrine (5%–10%) with different grades of severity (40). While our current therapy strategy mainly focuses on blocking the inhibition signals to improve effector function, these hyperfunctional T cells induce severe autoimmune damage. Although multiple inhibitory receptors have been investigated to identify T cell exhaustion (2), molecular markers and transcriptional signatures identifying bona fide dysfunctional T cells is still lacking. Thus, there is an urgent need to identify novel tumor-restricted receptors that specifically targe tumor cells, while avoiding or limiting responses in the periphery.

Tumor-specific CD8^+^T cells have imprinted characteristics of exhaustion in pre-malignant or early-malignant period of tumorigenesis (41). Moreover, the selection of immunotherapy targets should also take into account adverse effect in normal or peripheral tissues to limit autoimmune-related immunopathology.

### T Cell Exhaustion Is Not a Fixed State Whereby Progenitor Exhausted T Cells Give Rise to Terminally Exhausted T Cells

**Figure 1:**
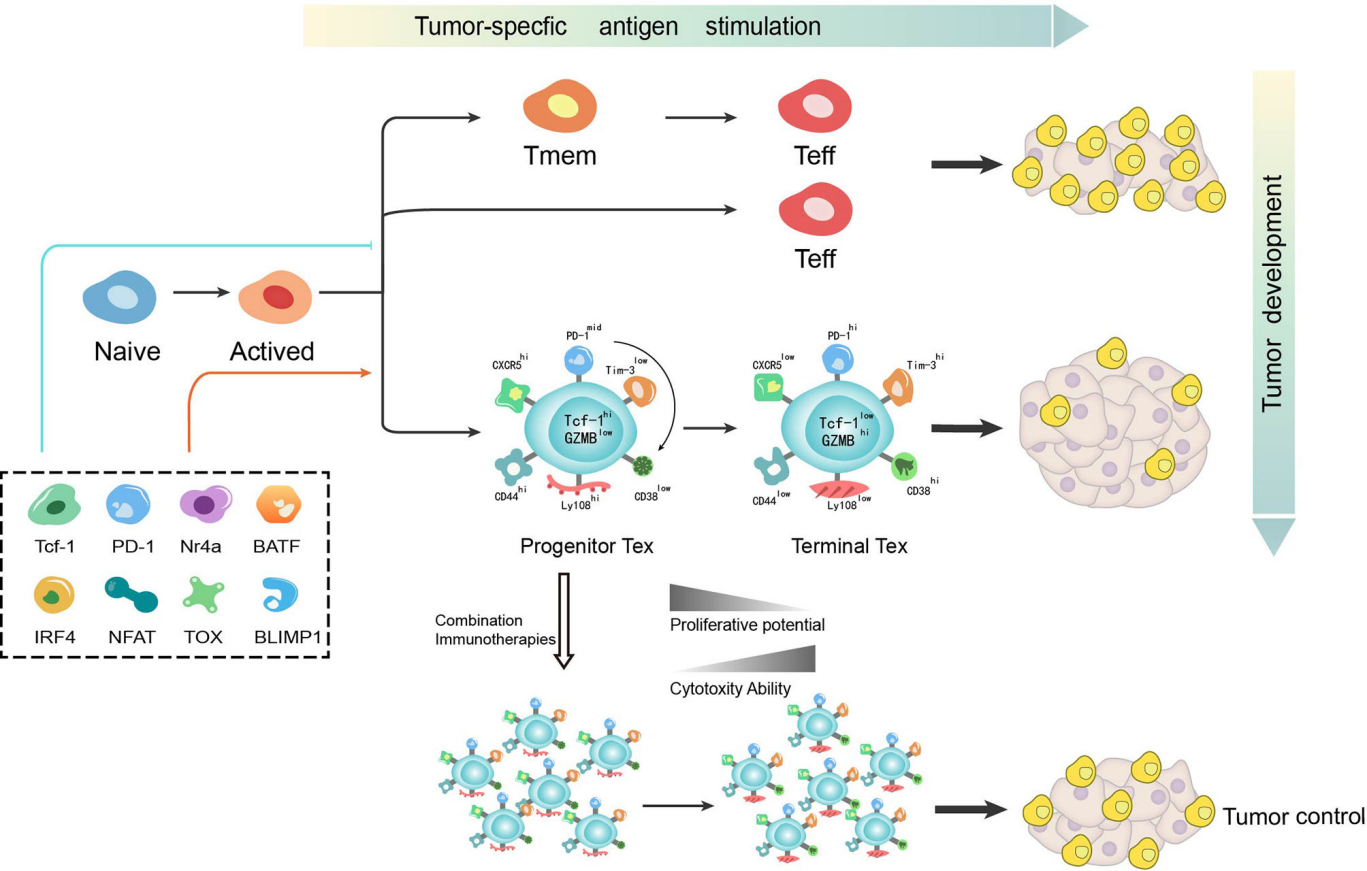
T cell differentiation is a dynamic process during which various mechanisms function to determine the bifurcation from effector or memory toward exhaustion. T cell exhaustion has recently been identified as not a fixed state whereby progenitor exhausted T cells give rise to terminally exhausted T cells. While progenitor exhausted T cells exhibit poor cytotoxicity but are long-lived with stem-like properties, terminally exhausted T cells have increased cytotoxicity but are short-lived. The distinct properties of exhausted T cells imply the potential of immunotherapy-based combination strategy in cancer treatment. Tmem, memory T cell; Teff, effector T cell; Tex, exhausted T cell.

Exhaustion has recently been identified as a dynamic process from “progenitor exhaustion” to “terminally exhaustion” during the process of chronic infection or cancer (42–45). (**Figure 1**) Several studies have been carried out to further recognize properties and related mechanisms of exhausted T cells.

During chronic LCMV infection, αPD-L1 blockade is found to selectively function on heterogenous exhausted T cells (46). While one subset that expresses intermediate levels of PD-1 and high levels of CD44 is reversed by αPD-L1 blockade, the other with high levels of PD-1 and intermediate levels of CD44 remains unresponsive (46). Using ATAC-seq in the LCMV viral model, some open chromatin regions are found to be “locked in” to a state unreversed upon immune checkpoint blockade thereby limiting efficacy (47). By analyzing the molecular and transcriptional characteristics of exhausted T cells, two distinct subsets of virus-specific exhausted T cells are found to cooperate to control chronic infection (48). Of interest here, this phenomenon is also found during tumorigenesis after the discovery in chronic infection (41, 49). Among exhausted CD8^+^ T cells in chronic infection or tumor microenvironment, a small population of the progenitor exhausted T cells retain stem-like properties and the major population, “terminally exhausted” TILs are characterized with high cytotoxicity (43, 50). While terminally exhausted T cells remain unresponsive to vaccination and checkpoint blockade immunotherapy, progenitor exhausted T cells can be transferred into a host with terminally exhausted T cells with increased cytotoxicity, but are short-lived (50, 51). By analyzing the chromatin state and surface markers of these two subtypes, the initial plastic state was also found to transit into a fixed state as terminal exhaustion in the context of persistent antigen stimulation, which is seen as elevated expression of CD38, CD101 and CD30L and low expression of CD5 with similar expression of PD-1 and LAG-3 (49). Thus, exhaustion is a dynamic process from progenitor exhaustion to terminal exhaustion in chronic infection or cancer, during which these two subsets respond differently to vaccination and checkpoint blockade.

Transcription factor T cell factor 1 (TCF-1, encoded by *Tcf7*) is a key transcription factor in progenitor exhausted CD8^+^ T cells during chronic infection and cancer (29, 42, 50–54). While TCF-1^+^PD-1^+^ CD8^+^ T cells are defined as “progenitor exhausted T cells” having expansion, regeneration and differentiation capacity, TCF-1^−^PD-1^+^ CD8^+^ T cells defined as “terminally exhausted T cells” are more exhausted but with increased cytotoxicity (29). Thus, TCF-1 is thought to play a significant role in differentiation of exhausted T cells. In chronic infection, TCF-1 is found to meditate bifurcation that represses development of a terminal effector but fosters progenitor exhaustion (55). To seed development of progenitor exhausted T cells in chronic viral infection, TCF-1 represses T-bet but promotes Eomes expression and drives c-Myb expression that controls Bcl-2 and survival. In addition, PD-1 is found to stabilize this TCF-1^+^ precursor cell pool (55). While PD-1^hi^ was previously regarded as an emblem for terminal exhaustion, the absence of PD-1 also leads to the accumulation of more cytotoxic but terminally exhausted T cells (30). Further, as PD-1 was also recognized as a promoter of terminal exhaustion, this discovery recovers its significant role in maintaining a durable immune reaction by inducing differentiation of progenitor exhausted T cells. One possible mechanism for the connection between PD-1 and TCF-1 is attenuation of TCR and/or CD28 signaling by PD-1 to prevent loss of TCF-1 expression (55). Another possibility is that a PD-1-BATF-TCF-1 feedback circuit exists in the precursor cell pool where BATF has been identified downstream of PD-1 and is positively correlated with TCF-1 in precursor cells (55). However, it has not yet to be elucidated whether TCF-1 in a tumor context also mediates the bifurcation of T cell differentiation from effector or memory toward exhaustion in the same mechanism. The discovery of an exhaustion induction mechanism in chronic infection provides a starting model to further understand exhaustion induction of tumorigenesis. Thus, there is still much to discover about the mechanism(s) accounting for the development of exhausted T cells in the tumor microenvironment.

Recently, in the chronic infection model, a transitory population marked by expression of CX3CR1 has been identified, which differentiated into the terminally exhausted T cells defined by upregulation of CD101 (44). The differentiation of this transitory population was attenuated by persistent antigen stimulation or suboptimally priming while PD-1 pathway blockade was found to expand the population (44, 56). However, more studies are demanding to further divide the subpopulation of exhausted T cells in cancer. Although rapid development in recognition of inhibitory receptors innovates tumor immunotherapy, more specific molecular investigation is needed to clarify the explicit stages of T cell differentiation. Considering the fact that T cell exhaustion is a dynamic process from progenitor to terminal exhaustion with distinct properties, more precise strategies of immunotherapy need to be discovered to intervene in the differentiation toward exhaustion and reverse certain stages of exhausted T cells.

## Dynamic Differentiation During Exhaustion Implies Combination Therapy Strategy

PD-1 pathway inhibitors have shown great success in cancer, especially in advanced melanoma, non-small-cell lung cancer, renal cell carcinoma and metastatic bladder cancer (4). However, this monotherapy approach is only effective in a subset of patients and partially responsive in the majority of patients (57–59). It is well acknowledged in the field that the clinical efficacy of immune checkpoint inhibitors is largely dependent on the density of pre-existing tumor-infiltrating CD8^+^ T cells (60). The baseline density and location of T cells is also essential for the success and durability of immune therapy (60), whereas CD8^+^ T cell infiltration does not appear to correlate with clinical parameters such as disease stage or patient age (61). However, the presence of terminally differentiated T cells, which derive from progenitor exhausted T cells as aforementioned, positively correlates with the total number of tumor-infiltrating T cells and prevents disease progression (51). Apart from the infiltration of T cells, tumor burden is found to determine severity of exhaustion and T cell reinvigoration by PD-1 pathway inhibitors in preclinical models and cancer patients (13, 62). As eluded to, T cell exhaustion is a dynamic process with variational properties from stem-like self-renewal toward terminally exhaustion. While progenitor exhausted T cells exhibit poor cytotoxicity but are long-lived with stem-like properties, terminally exhausted T cells have increased cytotoxicity but are short-lived. This phenomenon is consistent with previous observations that a complete immune response is required for both the effective ability to clear antigen and durable potency to deal with sustained antigen stimulation. As a compensatory mechanism for immune control, terminally exhausted T cells function as a significant but transient effector. Though PD-1 pathway inhibitors transfer stem-like progenitor exhausted T cells into cytotoxic terminally exhausted T cells to temporarily control the tumor (29, 50, 51),terminally exhausted T cells, with depletion of the progenitor T cell pool, finally fail to function in face of exorbitant tumor burden. These observations may help to explain the reasons for the failure of immune monotherapy. Therefore, the ratio of exhausted T cell reinvigoration to tumor burden is put forward as a predictor of clinical efficacy (63). Moreover, combination therapy with PD-1 pathway inhibitors needs to be urgently to be developed in clinical practice.

On the other hand, with further recognition of the complexity of TME, tumors are divided into four subgroups (including hot tumors, altered-immunosuppressed tumors, altered-excluded tumors and cold tumors) based on the CD8^+^T cell landscape within TME (64). In hot or altered-immunosuppressed tumors, the failure of immune monotherapy mainly results from TILs dysfunction, while ICB-based combination therapy mainly refers to improvement in T cell function (immune therapy targeting other co-inhibitory receptor such as CTLA-4, LAG-3, and Tim-3) or the removal of inhibition from immune suppression factors (such as the blocking pathway of T_reg_ or myeloid- derived suppressor cells). As for altered-excluded tumors, deficiency in T cell-recruiting signaling and physical barriers to T cell penetration from abnormal vascular structure results in the failure of T cell infiltration. Thus, combination therapy strategies in altered excluded tumors mainly focus on facilitation of T cell recruitment or angiogenesis inhibitors with ICB. Cold tumors lack of pre-existing immune response is due mainly to low immunogenicity and failed T cell priming. Thus, the most important strategy is to turn cold tumors into hot tumors. The priming therapy (including vaccines, chemotherapy or radiotherapy) enhances T cell response and PD-1 pathway inhibitors remove cancer-meditated suppression, which implies the rationality of combination immune-activating therapy with immunotherapy in cold tumors.

Thus, in clinical practice, the dynamic differentiation during exhaustion essentially limits the effectiveness of immune monotherapy in the context of exorbitant tumor burden. Besides, the various immune properties of tumor emphasize the significance of combination therapy. Hence, it is important to now consider ICB-based combination therapy focusing on the expansion of the progenitor T cell pool or the reduction of tumor burden, implying the rationality and current clinical practice of combination with other therapy strategy (**Table 1**).

**Table 1:** Intervention strategies based on the cancer-immunity cycle for different immune types of tumor.

Tumor classifications	Characteristics	Intervention processes	Combined strategies
**Hot immune tumors**	•High degree of T cell infiltration	•Killing of cancer cells	•Immune checkpoint blockades (e.g., anti-CTLA-4 and anti-PD-1 mechanism)
**Altered-immunosuppressed immune tumors**	•Poor T cell infiltration •High presence of tumor suppressive cells and inhibitory mediators	•Infiltration of T cells into tumors •Killing of cancer cells	•Anti-VEGF •Immune checkpoint blockades •Removal of immune suppression (e.g., blockades of CD39, CD73, IL-10 or TGF-β)
**Altered-excluded immune tumors**	•No T cell infiltration within tumor but accumulation at tumor borders •Aberrant tumor vasculature and/or stroma	•Infiltration of T cells into tumors •Killing of cancer cells	•Anti-VEGF •Immune checkpoint blockades
**Cold immune tumors**	•Absent T cell infiltration •Suboptimal T cell priming	•Release of cancer cell antigens •Cancer antigen presentation •Priming and activation •Infiltration of T cells into tumors •Recognition of cancer cells by T cells •Killing of cancer cells	•Immunogenic cancer cell death (chemotherapy, radiotherapy and targeted therapy) •Enhanced APC function (e.g., anti-CD40) •Vaccines •Anti-VEGF •CART •Immune checkpoint blockades

CTLA-4, cytotoxic T lymphocyte-associated antigen 4; PD-1, programmed cell death 1; VEGF, vascular endothelial growth factor; CART, chimeric antigen receptor T-cell immunotherapy.

### Combination With Vaccines

Cancer vaccination relies on the identification of putative antigen or antigenic epitopes and then transfer into patients through a variety of approaches, such as whole tumor cells, MHC-specific peptides, whole or partial proteins encoded by RNA or DNA, or in recombinant viral or bacterial vecto expressed in dendritic cells (DCs) (65). Though showing promise in clinical practice, cancer vaccination alone has not been very effective in several clinical trials (65). The immunosuppression of TME was considered to be the major barrier for a sustained immune response, which implies a combination strategy with ICB. On the other hand, mechanisms that do not allow T cell activation could result in immune therapy resistance (5). T cell priming is defined as the events that naïve T cells are initiated from a quiescent state to an activated state (66). PD-1 blockade in unprimed or suboptimally primed CD8^+^ T cells could induce dysfunctional PD-1^+^CD38^hi^CD8^+^ cells leading to further resistance, which can be reversed by proper antigen stimulation (67). While immunosuppression in the TME impedes vaccine-induced immune effectors, combination with PD-1 pathway inhibitors significantly improved the overall survival period. For instance, in a preclinical model, combination therapy has been found to increase the infiltration of memory precursor effector cells in canonical non-immunogenic tumors, such as breast cancer and pancreatic ductal adenocarcinoma (68, 69). It implies for a rationale combination of vaccines and immunotherapy.

There are currently two FDA-approved therapeutic cancer vaccines: sipuleucel-T and T-VEC. Sipuleucel-T functions as a DC vaccine using a recombinant of the prostate tumor-associated antigen prostatic acid phosphatase (PAP) and granulocyte-macrophage colony-stimulating factor (GM-CSF). Expression of PAP increases during prostate cancer progression, activating the immune system and GM-CSF sustains DC maturation. Sipuleucel-T was approved by the FDA in 2010 for men with asymptomatic or minimally symptomatic hormone refractory prostate cancer based on the phase III IMPACT trial (70). Men who received Sipuleucel-T had a median overall survival (OS) of 25.8 months versus 21.7 months with placebo (HR: 0.78; 95% CI: 0.61–0.98; p = 0.03). In a phase I study of ipilimumab plus Sipuleucel-T for prostate cancer, six of nine patients treated had medial survival surpassing 4 years (71). On the other hand, T-VEC is an intralesional oncolytic viral vaccine composed of a modified herpes simplex virus type 1 encoding GM-CSF, which was approved by the FDA in 2015 for patients with recurrent melanoma. In the T-VEC OPTiM trial and a phase III MASTERKEY-265 trial, the combination of T-VEC and pembrolizumab increased infiltration of CD8^+^ T cells, PD-L1 expression and interferon (IFN)-γ levels, and thus improved treatment efficacy (72).

Moreover, with the development of high-throughput screening techniques and epitope-predicting algorithms, novel personal targeted vaccines unique to each patient are being tested in several clinical trials. One study has demonstrated the feasibility, safety and immunogenicity of a personalized vaccine in 20 advanced melanoma patients. Of six vaccinated patients, four had no recurrence at 25 months after vaccination while two recurrent patients subsequently received anti-PD-1 therapy and experienced complete tumor regression with expansion of neoantigen-specific T cells (73). Another RNA-based poly-neo-epitope vaccine were used in 13 advanced melanoma patients and showed sustained progression-free survival and was well-tolerated (74). In addition, there is currently an ongoing clinical trial (NCT02897765) combining a personalized vaccine (NEO-PV-01) with anti-PD-1 in patients with advanced cancers including melanoma, NSCLC and bladder cancer. Interim analysis has shown great success with 68.8% of patients showing a partial response (PR) and 6.3% showing a complete response (CR) for melanoma and, 45.5% of patients showing a PR in NSCLC. What is more, an RNA vaccine targeting four non-mutated, tumor-associated antigens has been proved to induce durable response even in checkpoint-inhibitor-treated melanoma (75). The RNA vaccine, alone or in combination with ICBs, induced more infiltration of immune cells. At the meanwhile, tumor burden at baseline has also been associated with the final response. Thus, vaccines have shown great potential in priming immune cells and inducing the differentiation of progenitor exhausted T cells, which implies the rationality of combination with immunotherapy. Though achieving some success, the response could be obstructed by the immunosuppression of TME and the exorbitant tumor burden, implying more combination therapies to be further developed. As the success of combination of vaccine and immunotherapy, other immune-activating strategies are also worth for the further development.

### Combination With Chemotherapy

Conventional therapies such as chemotherapy and radiotherapy can directly reduce tumor burden, however, they have also been found to be associated immunological effects requiring further understanding of tumor immune microenvironment. Genotoxic chemotherapies (such as anthracyclines and oxaliplatin) induce mutations and elicit the release of tumor antigens which increases the immunogenicity of tumor (64). On the other hand, these therapies can also induce immunological cell death (ICD) and convert tumor into an *in situ* vaccine, leading to the release of damage-associated molecular pattern molecules (DAMPs), such as calreticulin, high mobility group box 1 (HMGB1) or adenosine triphosphate (ATP), which activate apoptotic or necroptotic pathways and reactive immune responses (76). in addition, chemotherapeutic agents such as cyclophosphamide, taxanes or paclitaxel can activate immunostimulatory signals, though in lack of ICD induction (77). While chemotherapy often serves as the first-line therapy in tumor treatment, relapse is often observed probably due to the secondary expansion of immunosuppressing cells, exhaustion of immune effector cells or the emergence of chemoresistant tumor clones (77), which supports the rationale to combine immunotherapy to enhance immune effects. In a phase II study in metastatic NSCLC, phased ipilimumab plus paclitaxel and carboplatin showed an improved efficacy (78). Another phase II study has also shown the success of phased ipilimumab plus paclitaxel and carboplatin in extensive-disease-small-cell lung cancer (ED-SCLC) (79). Thus, the success of vaccines implies the rationality for the combination with immune-activating agents. Chemotherapy obviously reduced the tumor burden and simultaneously functions as an *in situ* vaccine optimally prime T cells which may induce the expansion of the pool of progenitor exhausted T cells, which suggests the prospect of the integration of chemotherapy and immunotherapy.

### Combination With Radiotherapy

Similar with chemotherapy, radiotherapy can also modulate immune response in addition to its tumor-debulking property. Aside from ICD-related mechanisms as aforementioned, radiotherapy show great promise in treating metastatic lesions with its “abscopal effect,” which reflects the phenomenon that while ionizing irradiation cause localized tumor death, non-irradiated metastatic sites have also been regressed through immune-related mechanisms. Thus, radiotherapy even focused on a single metastatic lesion is considered as a powerful tool to induce tumor into an *in situ* vaccine and optimally prime T cell activation as well as reduce tumor burden, which suggests the important role in combination with immunotherapy.

In phase III of the PACIFIC trial, patients with stage III unresectable NSCLC received chemoradiotherapy in addition to durvalumab, leading to improved median progression-free survival (PFS) from 5.6 months to 17.2 months and a 2-year overall survival improvement from 55.6% to 66.3% (80). In another phase II study, patients with metastatic NSCLC received stereotactic body radiation (SBRT) on a single tumor site preceding pembrolizumab (81). Influenced by the PD-L1–negative subgroup, this trial did not meet the study’s primary endpoint criteria but there was improved overall survival and progression-free survival, encouraging the possibility of further research. A phase I study of multisite SBRT followed by pembrolizumab in metastatic solid tumor patients has also been performed to assess the efficacy of SBRT and checkpoint inhibitors in different advanced tumors (82). Although median PFS was 3.1 months with no detailed record for the initial and sustained response status, the abscopal response rate in any single nonirradiated target metastasis was 26.9%. Despite these outcomes, more trials are demanding to explore the dosage, timing and sequence for the combination of radiotherapy and immunotherapy.

### Combination With Targeted Therapy

Recent studies of targeted therapies against the MAPK and VEGF signaling pathways not only can precisely inhibit oncogenic pathways, but also have effects on host immune modulation through increasing tumor antigenicity and promoting T cell infiltration (83), which rationalized the combination of immunotherapies with targeted therapies. For most altered-excluded tumors, deficiency in T cell-recruiting signaling and physical barriers to T cell penetration from abnormal vascular structure results in the failure of T cell infiltration, while anti-VEGF targeted therapy augments intra-tumoral T-cell infiltration, potentially through vascular normalization and endothelial cell activation. Further investigation of endothelial cell alterations indicated that the combinations of agents that inhibit the PD-1 and VEGF signaling pathways were adapting vessels for effective lymphocyte trafficking (83). The exact mechanism for the expansion of progenitor exhausted T cell pool is undefined but the vascular disorder obstructing to further recruitment probably explains the depletion. There are several clinical studies combining targeted therapy with checkpoint blockade that have displayed clinical benefit. In advanced metastatic melanoma, the combination of bevacizumab and ipilimumab was safely tolerated with increased adhesion molecule and immune cell infiltration as well as overall good clinical responses (84). Similar results have also been observed in metastatic renal cell carcinoma (85). Further, the combination of regorafenib and nivolumab significantly improved the efficacy in treatment of gastric and colorectal cancer (86). Though these results encourage further exploratory studies, severe irAEs have also been observed and therefore pretherapeutic evaluation should be carefully assessed (87, 88).

### Combination With Cytokines

Several cytokines (such as IL-2, IL-8, IL-10, IL-15, IFN-α, CSF-1, etc.) contribute to regulate every phase of the cancer-immunity cycle, including T cell priming, differentiation, expansion and even the effector function in the TME (1, 89). Among those, IL-2 mainly functions to expand the T cell population and natural killer (NK) cells while several clinical trials are ongoing based on the combination of IL-2 and immunotherapy (89).

The reduction of IL-2 is one of the first markers during the process of T cell exhaustion, however, the expression of IL-2Rβ, which correlates to the expression of PD-1, is elevated in exhausted CD8^+^ T cells (90). Therefore, IL-2 pathway plays a significant and complex role in the efficacy of treatment. The single agent is limited due to its pleiotropic effects on immune systems or the severe side effects, suggesting the necessity of combination therapy (91). NKTR-214 is a recombinant IL-2 pathway agonist with polyethylene glycol which preferentially activates IL-2βγR (IL2βγ receptor pathway) to foster the proliferation and activation of CD8^+^ T cells and NK calls without expansion of T regulatory cells in the TME (92). Considering the elevation of inhibitory receptors, combination with immunotherapy is recommended which results in the infiltration of T cell function, replenishment of progenitor exhausted T cell pool and enhancement of regional immune response. NKTR-214 in combination with ICBs is being evaluated in several clinical trials. (NCT03138499, NCT02983045, NCT03282344 and NCT03435640) Combination of NKTR-214 and nivolumab has shown great response in naïve or pre-ICB-treatment advanced solid tumors in a phase 1 trial (93).

## Conclusions

T cell exhaustion emerges in face of overwhelming activation and is modulated by multiple mechanisms during tumorigenesis. While multiple and high expression of inhibitory receptors are related to severity of exhaustion (66), inhibitory receptors are also found to participate into the activation process and play a physiological role in system (22, 25). Thus, new molecular targets specific for exhaustion need to be further explored whereby autoimmune-related adverse effects can also be limited to localized lesions.

On the other hand, recent studies identify T cell exhaustion as a dynamic process from progenitor exhausted T cells to terminally exhausted T cells in the face of persistent antigen stimulation or PD-1 pathway inhibitors (41, 48, 50, 94). While progenitor exhausted T cells are poorly cytolytic but long-lived with stem-like properties, terminally exhausted T cells have increased cytotoxicity but are short-lived. This dynamic differentiation reflects the limitation of immunotherapy in exorbitant tumor burden due to short-term effector function and depletion of the pre-existing T cell pool. However, “the Cancer-Immunity Cycle” suggests that rationale combination strategy to reduce tumor burden, improve tumor antigenicity and facilitate T cell infiltration can successfully overcome the failure of immune monotherapy (1). With the further understanding of immune-modulating function of vaccines and conventional therapy such as chemotherapy, radiotherapy and targeted therapy, new combination strategies have been put forward and multiple trials are ongoing to test further efficacy. Though showing promise in treatment of advanced tumors, combination toxicity still needs to be investigated.

## Author Contributions

HH, FY, and WJ contributed to original draft preparation and editing. GW, XZ, QS, WZ, YL, and YD contributed to the revision of paper. YH and QL contributed to the completion of the figure and table. WJ, YH, and WH contributed to writing the paper. All authors contributed to the article and approved the submitted version.

## Funding

The work was supported by the National Natural Science Foundation of China (81802350) and Natural Science Foundation of Zhejiang Province (LY18H160018).

## Conflict of Interest

The authors declare that the research was conducted in the absence of any commercial or financial relationships that could be construed as a potential conflict of interest.
